# Pubertal Labial Fusion: A Case Study

**DOI:** 10.7759/cureus.63773

**Published:** 2024-07-03

**Authors:** Aishwarya Gupta, Sandhya Pajai, Aditi Singh Thakur, Abhinav Ahuja, Nitish Batra

**Affiliations:** 1 Department of Obstetrics and Gynaecology, Datta Meghe Institute of Higher Education and Research, Wardha, IND; 2 Department of Medicine, Datta Meghe Institute of Higher Education and Research, Wardha, IND

**Keywords:** labial adhesions, reproductive health, adolescent health, genital anomalies, labial fusion, puberty

## Abstract

Labial fusion, though rare, can present during puberty, or even adolescence leading to challenges in diagnosis and management. This case report offers a detailed examination of the clinical manifestation, diagnostic process, and therapeutic approach in an adolescent girl with labial fusion. This report emphasizes the importance of early intervention to improve patient outcomes for this complex medical condition.

## Introduction

Labial fusion, also known as labial adhesion, labial agglutination, or labial synechiae, is the fusion of either the labia majora or minora frequently around the clitoris [1]. It is typically found in young girls before puberty, but may also arise during adolescence. In this case report, we present a pubertal girl with labial fusion and discuss the critical aspects of her diagnosis, treatment, and outcomes. Painful or difficulty urinating and the passage of menstrual blood are indications that a patient might have labial fusion. For these reasons, early detection and treatment are important. The application of estrogen cream may help separate fused labia, or surgery is part of its treatment choices. In a few cases, a biopsy might be done to rule out other conditions. The treatment outcome depends on the severity of the condition and the patient’s age. With early diagnosis and treatment, most patients achieve a full recovery and can go on to lead a normal, healthy life.

## Case presentation

A 19-year-old unmarried female presented with persistent symptoms of burning micturition, difficulty passing urine, and pain while passing menstrual blood. She was a known case of mild congenital epidermolysis bullosa. Her menstrual cycles were regular, with an average flow. There was no other significant past or present history.

On examination, the girl was thin built with well-developed secondary sexual characteristics. There was no palpable pelvic or abdominal mass. On per vaginal examination, there was a complete fusion of the labia minora, covering the clitoris, urethra, and hymen.

A detailed medical history of the patient was taken, in which the patient reported recurrent urinary tract infections due to the underlying obstructive pathology. The physical examination also supported the history, providing evidence of the anatomical defect.

Routine laboratory investigations were done to evaluate for other endocrine abnormalities and rule out infectious etiologies. A complete blood count, liver function test, kidney function test, fasting and postprandial blood sugar, urine routine, and microscopy were within normal limits. Imaging showed a normal-sized uterus and ovaries with no collection in the vaginal cavity or intrauterine cavity. However, there was dilatation of the pelvicalyceal system.

**Figure 1:**
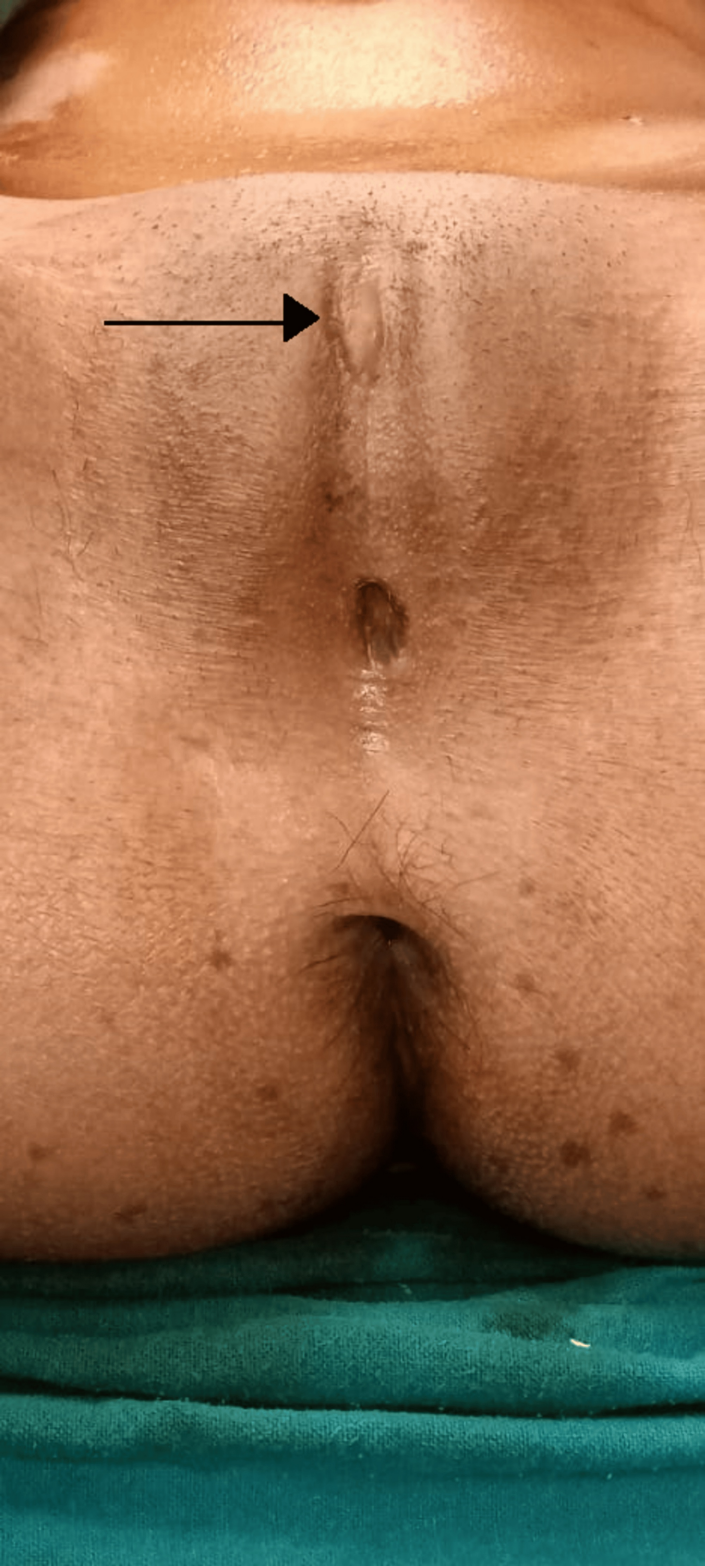
Complete fusion of labia minora covering the clitoris, urethra, and hymen. A small dimpling can be just above the anus marked by a black arrow.

**Figure 2:**
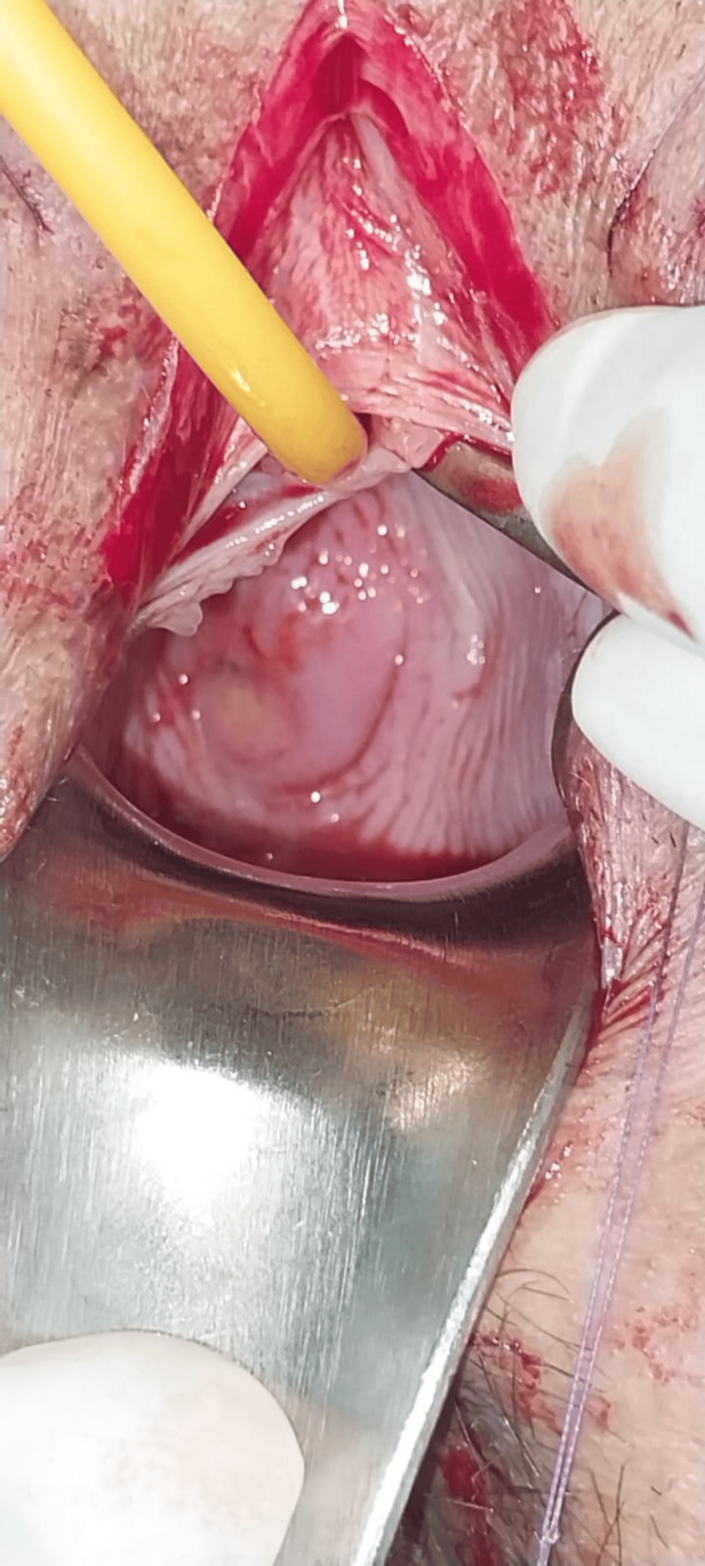
Visualization of the clitoris and urethra and successful catheterization of the bladder.

**Figure 3:**
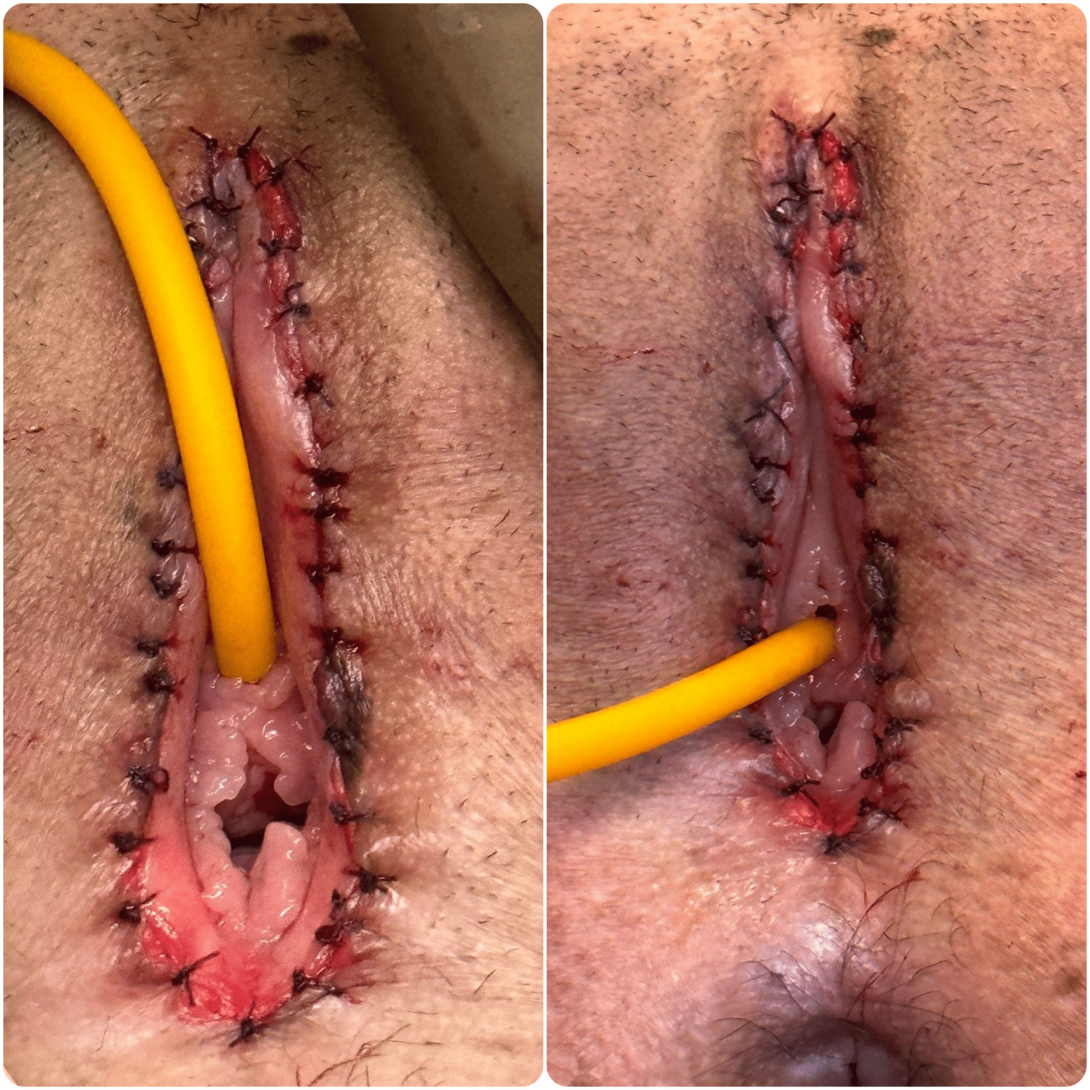
Reconstruction of the vulva using Vicryl 5-0.

The management plan included examination under anesthesia and vulval reconstruction. A small dimpling was noted 2 cm above the anus (Figure 1), where mosquito forceps were inserted and dissection was done. Once dissection was complete, there was complete visualization of the urethra, and the hymen and bladder catheterization was done (Figure 2). The reconstruction of the vulva was done using 5.0 Vicryl (Figure 3).

The patient was followed up postoperatively and there was substantial improvement in her symptoms. The catheter was removed on postoperative day three and the patient was discharged on postoperative day seven. There was an alleviation of discomfort and near-total improvement of urinary function. Complete resolution of labial fusion was observed during follow-up examinations and it served to correlate with the need for our multidisciplinary approach.

## Discussion

Pubertal labial fusion, characterized by the partial or complete adhesion of the labia minora, is rare in adolescent girls compared to younger girls. This discussion explores the diagnosis and treatment options of this condition [1]. The causes of labial fusion in pubertal girls are not well understood but may involve factors such as inflammation, irritation, hygiene practices, and genetic predisposition.

Unlike in prepubertal cases, low estrogen levels are less likely to be a contributing factor due to the hormonal changes typical of puberty [2]. Some cases of labial agglutination have been reported in the postpartum period, which is believed to be due to breastfeeding compounded with vaginal trauma during childbirth [1]. During breastfeeding, due to high prolactin levels, ovulation is suppressed and estrogen levels are decreased [3].

The condition can be challenging to diagnose due to the sensitive nature of its symptoms, which may include urinary difficulties, discomfort during physical activities, or visible changes in genital anatomy [4]. Labial fusion can be confused with several conditions, but thorough examination and history taking can help rule out other conditions such as imperforate hymen, characterized by a fully covered vaginal opening; Mayer-Rokitansky-Küster-Hauser syndrome, marked by the absence of the uterus and upper vagina; ureterocele, swelling at the ureter’s end; urethral prolapse, where the urethral lining protrudes; and vaginal atresia, where the vaginal canal is absent. This careful exclusion clarifies the diagnosis of labial fusion [5]. A careful physical examination is crucial for diagnosis. Treatment ranges from conservative approaches, such as topical estrogen or corticosteroid creams, to surgical intervention in more severe cases. Ongoing genital care and proper hygiene are important to prevent recurrence.

Labial fusion can significantly impact an adolescent’s body image and mental health, given the importance of this developmental stage for sexual and psychological identity [6]. Providing psychological support and promoting open discussions about the condition are essential. Pubertal labial fusion requires greater awareness among healthcare providers and the public. More research is needed to understand its etiology and improve management strategies, ensuring that affected adolescents receive timely and empathetic care.

## Conclusions

Labial fusion in pubertal girls warrants prompt recognition and tailored management to mitigate associated complications and optimize patient well-being. This comprehensive case report underscores the importance of a multidisciplinary approach, incorporating hormonal therapy and patient education, in the management of pubertal labial fusion. Early intervention and vigilant follow-up are essential for achieving successful outcomes and enhancing the quality of life for affected individuals.
